# The Metallome as a Link Between the “Omes” in Autism Spectrum Disorders

**DOI:** 10.3389/fnmol.2021.695873

**Published:** 2021-07-05

**Authors:** Janelle E. Stanton, Sigita Malijauskaite, Kieran McGourty, Andreas M. Grabrucker

**Affiliations:** ^1^Department of Biological Sciences, University of Limerick, Limerick, Ireland; ^2^Bernal Institute, University of Limerick, Limerick, Ireland; ^3^Department of Chemical Sciences, University of Limerick, Limerick, Ireland; ^4^Health Research Institute, University of Limerick, Limerick, Ireland

**Keywords:** autism, metallome, zinc deficiency, inflammation, microbiome, proteome, lipidome, transcriptome

## Abstract

Metal dyshomeostasis plays a significant role in various neurological diseases such as Alzheimer’s disease, Parkinson’s disease, Autism Spectrum Disorders (ASD), and many more. Like studies investigating the proteome, transcriptome, epigenome, microbiome, etc., for years, metallomics studies have focused on data from their domain, i.e., trace metal composition, only. Still, few have considered the links between other “omes,” which may together result in an individual’s specific pathologies. In particular, ASD have been reported to have multitudes of possible causal effects. Metallomics data focusing on metal deficiencies and dyshomeostasis can be linked to functions of metalloenzymes, metal transporters, and transcription factors, thus affecting the proteome and transcriptome. Furthermore, recent studies in ASD have emphasized the gut-brain axis, with alterations in the microbiome being linked to changes in the metabolome and inflammatory processes. However, the microbiome and other “omes” are heavily influenced by the metallome. Thus, here, we will summarize the known implications of a changed metallome for other “omes” in the body in the context of “omics” studies in ASD. We will highlight possible connections and propose a model that may explain the so far independently reported pathologies in ASD.

## Introduction

### The Metallome

Many metals are found in the human body. Although the essentiality is evident for K, Na, Ca, Mg, Fe, Zn, Cu, Mn, Mo, and Co, research is ongoing to decipher which other metals can be considered toxic, neutral, or beneficial to humans. The body’s metal composition varies between organs, tissue, and even on the subcellular level. Metals such as K, Na, Mg, and Ca are required in higher concentrations than trace metals such as Fe, Zn, Cu, and Mn. However, although trace metals may only be needed in small quantities, they are essential in metabolism, growth, and development (Zoroddu et al., 2019). Each metal may function in various processes as a cofactor for proteins such as enzymes and signaling ions. Some may also play a role in redox reactions accepting and donating electrons. Others play a structural role in biological molecules, while again others aid ligand binding to specific receptors (Mertz, 1998). The presence and concentration of each of the metals within a cellular compartment, cell, tissue, organ, etc., establishes the metallome. The metallome consists of “free” metal ions and metals in metal-containing molecules and proteins such as metalloproteins and metalloenzymes. Metal localization and levels are highly controlled by processes regulating metal homeostasis.

Metals with biological functions are also referred to as biometals. Each biometal has a network of transporters and buffering proteins involved in the uptake and transport that facilitate the process carried out by them. Notably, an imbalance of one particular trace metal is unlikely in disease states. Typically, the concentrations and ratios of multiple trace metals are impacted if one metal concentration is altered. An example of such an event is apparent in individuals with irritable bowel disease (IBD). Zinc concentrations are typically decreased from 15 to 40% in affected individuals. Conversely, copper concentrations are increased in these individuals (Schneider et al., 2020). This observation is in line with many studies, including research on ASD, that show that zinc and copper interact at the mucosal level and in the blood, where the ratios are inversely related (Li et al., 2014).

Metal homeostasis can be influenced by multiple factors such as genetic mutations but also by environmental factors such as nutritional intake, stress, and immune-modulatory events (Greenough et al., 2013; Grabrucker, 2016). Alterations in an individual’s metallome have been shown for the major neurodegenerative diseases such as Alzheimer’s and Parkinson’s disease, and Amyotrophic lateral sclerosis (ALS) (Scholefield et al., 2020). In the future, specific metal profiles may even be utilized as biomarkers of particular illnesses. They may be targeted to alleviate symptoms, and preventative measures may be put in place to counteract dyshomeostasis. In particular, biometals have gained attention in relation to ASD (Hagmeyer et al., 2018; Yang et al., 2019a; Nakagawa and Yamada, 2021).

### Metal Dyshomeostasis in Autism Spectrum Disorders (ASD)

Studies have consistently implicated the putative role of trace metals in neurobehavioral disorders and behaviors such as attention deficit hyperactivity (Fe, Zn), cognitive impairments (Zn, Fe, Mn, Co), depression (Zn, Cr, Fe, Co), and ASD (Zn, Cu, Co, Mn) (Cortese et al., 2012; Graham et al., 2014; Alghadir et al., 2016; Saghazadeh et al., 2017; Yang et al., 2019b). Specific behavioral features characterize ASD. Individuals with ASD display impaired social abilities, problems with language and communication, repetitive behaviors, and often experience sensory alterations and co-morbidities such as seizures, hyperactivity, sleep disorders, increased risk of allergies, gastrointestinal problems, and metabolic plus mitochondrial diseases, which commence early in life (Lord et al., 2020). ASD are a group of heterogeneous disorders that are likely caused and influenced by environmental and genetic factors or a combination of both. Thus, the complexity of ASD is difficult to address using animal models (Betancur, 2011). ASD may be characterized by elevated levels of mitochondrial dysfunction, lipid peroxidation, and decreased levels of antioxidants such as transferrin, ceruloplasmin, metallothionein, and glutathione which is evident through blood serum analysis (Chauhan et al., 2004). Accordingly, the mechanisms commonly studied in ASD in relation to metal dyshomeostasis include various factors such as oxidative stress, the competition of toxic metals with essential metals such as Cu and Zn, inflammation, neurotransmitter synthesis, and neuronal dysfunction, such as impaired synaptic function and neurogenesis (Vela et al., 2015; Ha et al., 2018; Pangrazzi et al., 2020). Metal dyshomeostasis in ASD has been consistently under review in recent years. Most frequently, an imbalance in the Zn:Cu ratio has been reported in ASD, and mechanistic studies hint at a causative role (Bjorklund, 2013).

For example, a decrease in Zn and an increase in Cu results in higher levels of oxidative stress. Oxidative stress is caused by the oxidation of biomolecules, leading to damage of cells and tissues and is contributing to inflammation in many diseases (Singh et al., 2019a). Reactive oxygen species (ROS), including H_2_O_2_ and OH^–^, are continuously present under aerobic conditions. Particularly in the case of increased Cu and decreased Zn concentrations, the positive regulation of apometallothionein is affected. Apomethallothionein binds with a high affinity to toxic metals and, as such, plays a pivotal role alongside Zn in the detoxification of toxic metals (Kang, 2006; Macedoni-Lukšič et al., 2015; Mostafa et al., 2016). Metals such as Cd, Pb, and Hg have been implicated in promoting intracellular ROS production. However, not all metals can act as electron donors or acceptors. For example, Zn is redox inert and considered an antioxidant through indirect interactions, acting as an inhibitor of NADPH oxidases which catalyzes the production of O_2_ using NADPH as an electron donor (Marreiro et al., 2017). In oxidative stress conditions, superoxide dismutase (SOD), which contains both Cu, Mn, and Zn, converts O_2_ to H_2_O_2_ (Cortese et al., 2008). A deficiency in either Zn, Cu, or Mn have been shown to impact oxidative stress by decreasing the levels of SOD (Meguid et al., 2011). Zn, Cu, and Mn also increase glutathione production, which maintains the cellular redox state acting as a further protective function in oxidative stress. Evidence suggests that toxic metals such as Cd contribute to oxidative stress, thereby promoting inflammation by mediating the activity of the transcription factors NF-κB and AP-1, which are redox-sensitive (Yang et al., 2007). ROS influence the pathophysiology of various neurobehavioral disorders, but an imbalance of these antioxidant enzymes has been particularly shown to correlate with regressive ASD (Chauhan et al., 2004).

ROS production is closely linked to pro-inflammatory processes. Zinc can downregulate the production of inflammatory cytokines through NF-κB signaling (Foster and Samman, 2012). Throughout fetal development, zinc-deficient conditions result in increased inflammation and altered central nervous system (CNS) development (Sauer et al., 2016). Increased inflammation has been reported in many human and animal studies on ASD (Theoharides et al., 2016; Freitas et al., 2018).

Free Zn in the CNS is contained within various brain regions, with the highest concentration found in the hippocampus. It is then released into the synaptic cleft to regulate the activity of multiple postsynaptic receptors such as N-methyl-D-aspartate receptors (Chowanadisai et al., 2005; Stoll et al., 2007). Zinc may enter the postsynaptic compartment and is also released into the postsynapse from intracellular zinc stores in a synaptic-activity-dependent manner. Postsynaptic scaffold formation is a pivotal function of Zn mediated by proteins such as SHANK2 and SHANK3. These proteins are at the center of an ASD-linked postsynaptic signaling network (Grabrucker et al., 2014).

Also, neurogenesis is controlled by metal ion concentrations such as Fe, Cu, and Zn. In ASD, it is typically hindered due to effects in various pathways (Wang et al., 2019; Kumar et al., 2021). Zn deficiency has been shown to reduce the proliferative development of neuronal stem cells, leading to decreased cell survival (Pfaender et al., 2016). Even marginal Zn deficiency affects neurogenesis by altering the number of neurons and reducing neuronal specification (Kumar et al., 2021). Altered neurogenesis has also been implicated in ASD. Taken together, due to the various roles of metals in human physiology, the altered metal composition may have significant knock-on effects on many other processes that control proteostasis, gene expression, cell signaling, organ development, and metabolism.

### A Link Between the Metallome and Other “Omes” of the Body

Since metals occur in the human body as charged elements and ions cannot diffuse passively through biological membranes, their transport and storage are mediated by many proteins. Indeed, it has been estimated that 10% of the human genome encodes for zinc-binding proteins (Andreini et al., 2006). Therefore, if genetic mutations develop that alter the protein function of metal-binding proteins, they may influence the metallome and lead to an imbalance of metals in an individual (Zhang et al., 2019). Through this mechanism, the transcriptome (the set of all coding and non-coding RNA transcripts at a particular time in a specific location) and proteome (the set of proteins present at a specific time in a certain location) are tightly linked to the metallome. However, metal-binding proteins are found among the class of DNA transcription factors, DNA repair pathways, and proteins and enzymes regulating cellular signaling pathways resulting in the production or degradation of selected transcripts/proteins (Lin et al., 2006). Thus, the metallome also affects the genome, transcriptome, and proteome through various mechanisms.

A large proportion of studies have turned to research on the gut-brain axis in various neurobehavioral disorders. Through this, insights into how the gut microbiome influences the brain and neural signaling have been emerging (Mayer et al., 2014). Microbiomics comprises the investigation of microbial compositions within an individual at a certain time, specifically within different organs/tissues within individuals, such as the gut (Gilbert et al., 2018). The gut microbiome can influence gastrointestinal (GI) physiology along with inflammation and immune function (Vuong and Hsiao, 2017). Additionally, the microbiome has been thought to play a role in the inflammasome simultaneously. The inflammasome comprises an assembly cascade of immune cell inflammatory response cytokines such as NLRP’s (Nucleotide-binding oligomerization domain, Leucine rich Repeat and Pyrin domain containing), caspases, and interleukins (ILs) (Zheng et al., 2020). For example, the regulation of IL-18-mediated antimicrobial peptide production by the gut microbiome has been shown (Chen, 2017). The inflammasome may also be controlled by epigenomic effects, whereby post-translational modifications such as phosphorylation and histone ubiquitination affect many functions carried out by the inflammasome (Man and Kanneganti, 2015).

However, the microbiome, inflammasome, and epigenome (the chemical changes to an organism’s DNA and histone proteins) are also tightly linked to the metallome. Microbiota have needs for biometals, and the availability of these will influence the microbiota composition. For example, Zn deficiency promotes dysbiosis of the microbiota by favoring Enterobacterial colonization (Byndloss et al., 2018). On the other hand, microbiota composition will also affect the amount of trace metals available for the host, thereby affecting the metallome. The epigenome is also influenced by metal availability. The accumulation of metals such as Cd, Zn, and Cu have been shown to play a role in altering epigenetic outcomes, including DNA methylation, histone modification, and miRNAs (Mordaunt et al., 2019; Brito et al., 2020). Examples include the methylation of Metallothionein-1 (MT-1), whereby Cd changes the methylation patterns of the Hpa II sites within the *MT-1* gene, which expression decreases in return (Compere and Palmiter, 1981; Wang et al., 2012), and alterations in the promoter region of metallothionein-2 (MT2) through changing histone modification in mice in response to Zn deficiency (Kurita et al., 2013). As previously mentioned, reactive oxygen species and oxidative stress are influenced by metal availability, which has been shown to play a role in the promotion and inhibition of inflammasome activation, particularly the NLRP3 inflammasome (Martinon, 2010; Chen et al., 2016; Fan et al., 2017). In addition, a close link between inflammatory processes and microbiota composition exists. In line with this, zinc deficiency resulted in altered microbiota composition and increased inflammation in a mouse model (Sauer et al., 2019).

The proteome, metallome, and microbiome are also key determinants of the metabolome (the complete set of small-molecule chemicals found within a biological sample at a certain time). Metabolomic research in ASD primarily focuses on small-molecule metabolites in the brain, plasma, and urine (Kurochkin et al., 2019; Needham et al., 2021). So far, it has been reported that the metabolome of an individual corresponds to the microbiome of the individual. However, the impacts of metallome dyshomeostasis have rarely been monitored in parallel with both the gut microbiome and fecal metabolome. Through its interaction with the proteome and microbiome, major effects of the metallome on the metabolome are expected that may play a role in neurodevelopmental disorders (Mounicou et al., 2009; Grabrucker, 2020a). For example, exposure to chronic lead (Pb) has been shown to alter gut microbiota composition (microbiome), along with changes in the expression of genes (transcriptome/proteome) related to lipid metabolism and significantly altered metabolite concentrations (metabolome) in mice (Xia et al., 2018).

The lipidome (the totality of lipids present at a certain time in a specific location) and glycome (the entire complement of sugars present at a certain time in a specific location) are subsets of the metabolome (Santoru et al., 2017; Zierer et al., 2018; Kurochkin et al., 2019). Previous studies have focused on the effects of an altered metal availability on the lipidomic profiles and subsequent impact on inflammation. Such an example includes a recent study demonstrating a high-fat diet in mice and a Zn sufficient diet, leading to a highly protective profile in both liver and plasma lipoproteins and a greater antiatherogenic response in the liver when compared to Zn deficient mice (Kostara et al., 2018). Further to this, another study using rats focused on the effects of excess Cd. Upon exposure to excess Cd, an increase was observed in hepatic cholesterol, phospholipids, and triglycerides (Sarmiento-Ortega et al., 2017). These studies suggest a close connection between the metallome and lipidome.

## Discussion

### “Omics” Approaches for Understanding Pathomechanisms of ASD

The vast majority of ASD-related studies based on “omics” approaches focus on a single “ome” that is evaluated and used to make predictions about possible pathomechanisms (Supplementary Table 1). However, as described above, the “omes” of the body are interlinked, whereby alterations in one field may affect numerous others.

#### Metallomics

Studies of cellular metal compositions and their associated proteins have been of increasing interest throughout recent years. In these studies, various factors have been considered, such as age, the severity of ASD, impacts of toxic metals on essential metals, and the presence of specific metalloproteins such as metallothioneins (Vergani et al., 2011; Al-Farsi et al., 2013; Macedoni-Lukšič et al., 2015; Hawari et al., 2020). However, since the direct measurement of metal concentrations in brain tissue of human participants with ASD is currently not achievable, most metallomics studies were performed using blood, hair, nail, or tooth samples. Unfortunately, the focus on the ASD population is mostly neglecting the crucial developmental window where trace metal abnormalities have most impact, which may be *in utero*, with only a few studies being able to measure the metallome during pregnancy. Despite the importance of metal homeostasis in development and neurological functions, few metallomics studies have been performed in animal models for ASD, which would allow a direct assessment of metals in the brain tissue. For example, while hypozincemia was detected in the maternal immune activation (MIA) rat model (Galvão et al., 2015), to our knowledge, no metallomics data is available from other rodent models for ASD despite zinc supplementation being able to rescue some ASD behaviors in MIA mice, valproic acid-treated rodent models, and *Shank3* knockout mice (Grabrucker, 2020b).

However, some insights can be gained from epidemiological metallomics studies in ASD. For example, a metallomics study with 77 pairs of mothers and babies confirmed the relationship between the presence of toxic metals and levels of essential metals. The results demonstrated an accumulation of three specific toxic metals, lead, cadmium, and aluminum which decrease with age while showing a correlation between a deficiency in Zn and Mg and toxic metal accumulation in the offspring (Yasuda et al., 2020). This pattern, an increase in toxic metals, and a decrease in essential metals has been shown in a plethora of metallomics data. Many studies have shown that children with ASD display a higher body content of toxic metals such as Pb, Hg, and Cd compared to neurotypical controls (Cohen et al., 1982; Eppright et al., 1996; Filipek et al., 1999; Al-Ayadhi, 2005; Fido and Al-Saad, 2005; Nataf et al., 2006; Adams et al., 2007; Geier and Geier, 2007; Clark et al., 2010; Geier et al., 2010, 2012; Blaurock-Busch et al., 2011; Elsheshtawy et al., 2011; Lakshmi Priya and Geetha, 2011; Al-Farsi et al., 2013; Yasuda et al., 2013; Saghazadeh and Rezaei, 2017), and altered levels of essential metals, such as a decrease in Zn (Wecker et al., 1985; Blaurock-Busch et al., 2011; Elsheshtawy et al., 2011; Yasuda et al., 2011; Li et al., 2014; Tabatadze et al., 2015) and increase in Cu (Blaurock-Busch et al., 2011; Elsheshtawy et al., 2011; Lakshmi Priya and Geetha, 2011; Russo and deVito, 2011; Li et al., 2014; Tabatadze et al., 2015). In most studies, both a deficiency in Zn and an increase in Cu occur in the same samples. However, the results are sometimes inconsistent, and the quality of studies differs due to sample size, a study population that is limited to a specific region, differences in protocols such as measuring fasting vs. non-fasting blood samples, and methods such as using inductively coupled plasma mass spectrometry (ICP-MS) or atomic absorption spectroscopy (AAS). In addition, participants with ASD may be selected based on an independently obtained diagnosis, or more carefully assessed within a study, for example, regarding their autism severity. Nevertheless, three meta-analyses investigating a total of 19 different case control studies with ca. 1,516 participants (831 ASD, 685 controls) confirm that low Zn levels are associated with ASD (Sayehmiri et al., 2015; Babaknejad et al., 2016; Saghazadeh et al., 2017).

The probably most extensive study to date that investigated 1,967 hair samples of individuals with ASD found a clear correlation between age and abnormal metal levels (Yasuda et al., 2011, 2013). Intriguingly, in several studies, the severity of trace metal abnormalities was directly correlated to the severity of ASD symptoms (Elsheshtawy et al., 2011; Lakshmi Priya and Geetha, 2011; Li et al., 2014).

Although a recent meta-analysis investigating iron levels in individuals with and without ASD found no significant differences between the two groups (Tseng et al., 2018), a Chinese cohort study with 254 children that investigated whole blood trace metal levels demonstrated a significant difference between individuals with ASD and without, with a 5% higher Fe concentration in boys with ASD when compared to girls with ASD (Wu et al., 2019). A previous study had found similar differences using hair samples where a significant increase in Fe concentration was found in boys but not girls. Future studies should focus on the mechanism of this gender difference and evaluate whether the Fe level may be a suitable predictor of ASD in boys (Bener et al., 2017; Skalny et al., 2017). In addition, future studies will benefit from the correlation of metal abnormalities to specific features and severities of ASD symptoms in male and female participants (Fiore et al., 2020). Overall, to date, several high-quality studies that use ICP-MS, more than 100 participants, and correlate metallomics data with ASD severity (Russo and deVito, 2011; Arora et al., 2017) could link abnormal metal levels to ASD, despite some variability due to the various factors influencing the results of metallomics studies such as the age of the participants, diet, method of assessment, biosample used, and heterogeneity of the ASD cohort. From the studies and meta-analyses performed, abnormal Zn/Cu ratios consistently emerge as being linked to ASD. The detection of altered Cu/Zn ratios has been proposed as a biomarker for ASD (Faber et al., 2009). It may be complemented in the future by detection of other dysregulated metals defining an ASD-specific metal profile. Evaluating metal profiles has the advantage that early detection, and therefore, early intervention, may be possible as studies have shown that metal alterations are most prominent in children with ASD under the age of three (Yasuda et al., 2011) thus before most ASD diagnoses are established. In addition, detection of these metal profiles during pregnancy may not only allow intervention but even prevention.

#### Proteomics/Transcriptomics

Proteomic/transcriptomic studies in ASD investigate specific changes for the discovery of biomarkers or the identification of pathways that may contribute to the pathological features of ASD, such as oxidative stress, inflammation, and altered synaptic development and plasticity (Abraham et al., 2019b). Biomarker discovery is mostly made in human studies using easily accessible samples such as saliva, blood, and urine. In contrast, research on the pathomechanisms of ASD is mostly done with the help of cell culture and animal models, with few human studies using post-mortem cerebral tissue (Gandal et al., 2018; Schwede et al., 2018). These studies identified several significant differences in the proteome of individuals with ASD and model systems. For example, numerous genes/proteins were identified changed in these studies that have been implicated in metabolic functions, vesicular biology, mitochondria, and intracellular signaling. Pathway analysis using proteomics/transcriptomics data has also pointed to synaptic processes with significant alterations in glutamate (NMDA) receptor signaling identified in various brain regions. In addition, pathways involved in redox mechanisms and inflammatory processes were in the focus (Voineagu et al., 2011; Feng et al., 2017; Gandal et al., 2018; Schwede et al., 2018; Abraham et al., 2019a; Gordon et al., 2021; Hewitson et al., 2021). However, there is a lack of overlap between the genes/proteins identified due to the heterogeneity of ASD and different cohorts of varying ages and severity of symptoms used in human proteomic/transcriptomics studies (Wetie et al., 2015b; Cortelazzo et al., 2016). Major gene/protein alterations identified throughout the studies mentioned in Supplementary Table 1 include interleukins and inflammatory cytokines, myelination, mitochondrial, and synaptic proteins (Broek et al., 2014; Wetie et al., 2015a; Feng et al., 2017).

Transcriptomic analysis of both ASD mouse models and human postmortem brain tissue has additionally revealed the dysregulation of genes primarily involved in cellular stress responses, apoptosis, chromatin modification, and DNA repair. One such example includes 8-oxodeoxyguanosine, a heavily studied oxidative stress marker (Dizdaroglu et al., 2002). The increased expression is due to the inhibition of the Ogg1 protein through various possible mechanisms such as epigenetic histone modification or hypomethylation of the promoter sequence (Shpyleva et al., 2014). As previously mentioned, Zn and Cd dyshomeostasis are implicated in oxidative stress, contributing to the epigenetic mechanisms disrupting the Ogg1 expression (Karoui-Kharrat et al., 2017; Elwej et al., 2018). Several animal studies have been performed using *Shank3* knockout mice, BTBR mice, NMDA receptor subunit NR1 knockdown mice, or FMR1 knockout mice. These studies connect the synaptic disruptions with an altered cortical expression of proteins that function in mRNA transport, neurotransmission, and synaptic plasticity (Liao et al., 2008). Another study used the MeCP2-deficient mouse model to mimic Rett syndrome, revealed alterations in cholesterol synthesis and metabolism (Pacheco et al., 2017). Data analysis of the transcriptome, in particular, found varying mRNA co-expression modules along with distinct miRNA expression patterns among different cohorts of individuals with ASD when compared to neurotypically developing individuals (Ramaswami et al., 2020). Network approaches used in the transcriptomic analysis can deduce functional roles of genes based on the accompanying network. For example, module M12 included the overrepresentation of various ASD susceptibility genes such as *AHI1* and *SLC25A12* (Voineagu et al., 2011). In general, proteomics and transcriptomics studies in ASD hint at abnormalities of synaptic proteins, proteins regulating mitochondrial function, and proteins mediating immune processes, as well as lipid metabolism (Abraham et al., 2019b).

#### Epigenomics

Epigenomic studies in ASD have a focus on fetal development. Findings suggest an important role for epigenetic alterations in regulating the transcriptional level of gene modules (co-expressed genes) (Amiri et al., 2018). Epigenomic studies in ASD primarily focus on methylation and acetylation patterns commencing *in utero*. Various ASD-associated genes and receptors have been identified as the target of epigenetic alterations, such as *SHANK3, MECP2, FMR1*, etc. (Edamura and Pearson, 2005; Zhu et al., 2014; Yuksel et al., 2016; Lu et al., 2020). A histone acetylome-wide association study published in 2016 carried out ChIP-sequencing on 257 postmortem samples from individuals with ASD along with matched controls indicated that approximately 68% of ASD cases contained a mutual acetylome signature. Despite the heterogeneity of ASD etiology, acetylation patterns across cortical regions display similar alterations leaning toward collective downstream effects to the acetylome (Sun et al., 2016). Again, synaptic processes are targeted by many epigenetic modifications. For example, GRB10 contributes to a negative feedback loop that decreases mTORC1 signaling, contributing to ASD pathology through synaptic excitation and regulation. Thus, the deacetylation of GRB10 may influence a common synaptic pathway, thereby contributing to idiopathic ASD (Sun et al., 2016). A meta-analysis using blood samples carried out on 800 individuals with ASD suggested 55 significant CpG sites (Andrews et al., 2018). The major findings concluded from epigenomic studies show major hypomethylation and hypermethylation at specific loci in neuronal signaling and developmental genomic regions. Such an example includes the hypermethylation of the *GAD1* promoter leading to an increase in promoter binding of MeCP2, which has been found in both human and relevant animal models in neurobehavioral disorders (Zhubi et al., 2014). This hypermethylation, in turn, has been linked further to prenatal immune activation (Labouesse et al., 2015).

Further research has indicated that dysregulation of microRNAs in the brain contributes to ASD pathogenesis. A study has found both hypomethylated and overexpressed microRNAs and the respective genes in ASD brain samples such as miR-142, miR-451a, and miR-144-3p which have been shown to play a role in synaptic function and also target the oxytocin receptor gene (Mor et al., 2015).

#### Metabolomics (Lipidomics, Glycomics)

In humans, alterations in the metabolome of individuals with ASD have been assessed mainly through blood and urine samples. A study focused on gray matter in the prefrontal cortex and its metabolite composition has identified 16 altered metabolic pathways, including cysteine and methionine metabolism, glutathione metabolism, citric acid cycle, and various others, whereas urine and blood sampling identified 10 alterations such as purine and pyruvate metabolism, nicotinate and nicotinamide metabolism and galactose metabolism (Kurochkin et al., 2019). A recent study aimed to characterize metabolomic features in hyperpurinergic conditions using a maternal immune activation mouse model. Fifty percent of plasma metabolites (202/401) from 37 pathways were altered significantly, with xanthosine, dopamine, and L-isoleucine ranked as the most altered metabolites in the maternal immune activation mice (Zolkipli-Cunningham et al., 2021). A further study has focused on valproic acid-induced ASD rodent models. Particularly in the hippocampus, significant alterations were observed as prematurely as neonatal development, whereby 16 metabolites differed significantly, including glutamate, uracil, and N-acetylaspartate (Abreu et al., 2021). Another quantitative metabolomics study was carried out on mice. A total of 999 lipids were identified, where 13.7% showed a significant difference between ASD and typically developing controls. The most prominent lipids included glycerolipids, cholesterol, poly-unsaturated fatty acids, and phospholipids, where short-chain fatty acids were found in a lesser quantity in ASD (Needham et al., 2021). The overall findings reported in multiple studies are conflicted in terms of metabolomic profiles and gender. Further research will be required due to the inconsistency of the results (Ruoppolo et al., 2015; Diaz et al., 2016; Abreu et al., 2021; Courraud et al., 2021). Interestingly, alterations observed in infants from day 1 to 10 post-birth have been hypothesized to be linked to the microbiome and inflammasome (Courraud et al., 2021).

#### Microbiomics

Microbiome analysis using human samples and rodent models has consistently revealed significant differences between individuals with ASD and the control group. However, a lack of consistency is observed regarding the specific microbiota profile of the ASD group. Many changes are found on a bacterium phylum level rather than a species level. However, microbiome analysis may play a significant role in the characterization of subgroups within the ASD population (Finegold et al., 2010; Golubeva et al., 2017; Sauer et al., 2019; Sgritta et al., 2019). Gut dysbiosis plays a substantial role in the pathology of ASD. A particular emphasis has been put on the microbiome in the early development of individuals and the factors which may influence the gut flora, including antibiotic use, nutrition intake, infections, stress, etc. (Sivamaruthi et al., 2020). A focus on the treatment and reintroduction of bacterial strains has shown promising results in animal studies, particularly in mice. Such an example includes the treatment of *L. reuteri*, whereby ASD-like symptoms were diminished and reversed (Buffington et al., 2016). A similar approach was used in germ-free mice, which decreased abnormal behavior once they administered human “infant type” *Bifidobacterium* species (Luk et al., 2018). Further studies and meta-analyses have focused on bacterial composition and ratios, finding an increased ratio of *Firmicutes* to *Bacteroidetes* in ASD cohorts compared to typically developing cohorts (Borre et al., 2014). *Candida* has been found in a significantly higher abundance, while *Streptococcus, Prevotella*, and *Veillonella* are significantly decreased compared to individuals without ASD (Kushak et al., 2017; Strati et al., 2017). However, most meta-analyses in human and mouse models failed to reveal ASD-specific signatures despite most studies showing significant differences in microbiota composition of individuals with ASD.

#### Inflammasomics

Immune responses in ASD are often altered and may play a prominent role with regard to GI issues and other symptoms experienced by individuals with ASD. Recent studies show that inflammation is highly linked to the symptoms and severity of ASD in the individual (Patel et al., 2018). NLRP3 and AIM2 inflammasomes have been implicated in this process (Saresella et al., 2016). The dysregulation of several cytokines is further involved in the pathology of ASD (Zhu et al., 2006; Raison et al., 2010). Alterations in inflammatory levels result from multiple systemic and external factors that affect immune activation, such as maternal immune activation and allergies, autoimmunity, genetics, and maternal exposure to toxic metals (Crespi and Thiselton, 2011; Wei et al., 2011; Garay et al., 2013; Frye, 2015). Recent reviews have focused on cytokine expression measured from neonatal blood samples and cerebral spinal fluid, whereby alternative cytokine levels have been identified across different studies. A consistent finding includes the increased expression of IL-6 and tumor necrosis factors (TNF) (Ashwood et al., 2011; Abdallah et al., 2013; Tsilioni et al., 2015; Abruzzo et al., 2019). Conversely, various other studies have found conflicting data on other cytokines using different sample types, potentially indicating an impact of age, gender, and severity of ASD in the individuals (Abdallah et al., 2012; Masi et al., 2017; Siniscalco et al., 2018; Saghazadeh et al., 2019).

### Lessons From the Links Between “Omes” for ASD

**FIGURE 1:**
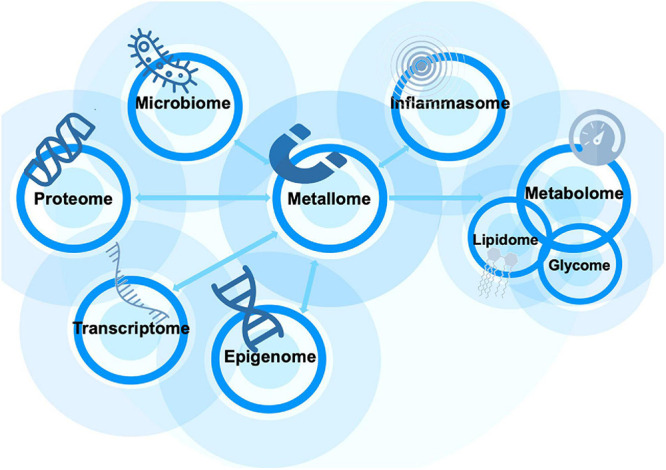
The metallome’s role in ASD can be expanded through its interactions with various other “omes.” Each arrow indicates a significant relationship whereby dyshomeostasis and further alterations in one impact other “omes” functionality.

Given that most “omes” are interlinked (Figure 1), it may be possible that the independently reported alterations indeed are part of one specific process that affects all “omes” of the body and that forms the biological core of the ASD pathology and reveals an ASD signature.

In ASD, metal dyshomeostasis will affect the proteome, epigenome, microbiome, metabolome, and inflammasome. Together, directly or indirectly, the changes in these “omes” will affect biological targets resulting in the pathologies underpinning ASD. For example, zinc deficiency during pregnancy alters microbiota composition and increases inflammatory marker expression in the periphery but also in the brain (Sauer and Grabrucker, 2019). Thus, alterations in the metal profile will have indirect effects on brain functions by causing extracerebral changes. However, systemic zinc deficiency will also affect brain zinc levels. Therefore, zinc-dependent proteins within the brain will directly react to differences in metal concentrations, as shown in *in vitro* experiments (Grabrucker et al., 2014).

Among individuals with ASD, synaptopathies have been identified as a prominent feature (Keller et al., 2017). Synaptic pathologies include impaired synapse formation and maturation as well as plasticity, probably mediated by the dysregulation of glutamatergic ion channels, alterations in various pre-and postsynaptic scaffolding and cell adhesion proteins (e.g., Neurexin, Neurologin, SHANK3), and proteins that function in intracellular signaling (e.g., PTEN, mTOR) (van Spronsen and Hoogenraad, 2010; Yoo et al., 2014; Xing et al., 2019). In line with this, transcriptomics, proteomics, and epigenomic studies have identified various synaptic proteins altered in ASD.

Metals will also affect synaptic processes through their role as cofactors in enzymatic functions, causing and reducing oxidative stress and functioning as intracellular signaling ions and neurotransmitters. Zn deficiency, for example, reduces synaptic protein scaffold formation through direct binding to SHANK3 and modulates NMDA and AMPA receptor function (Grabrucker, 2014; Grabrucker et al., 2014; Li et al., 2018a). Furthermore, Zn may also modify epigenomic alterations carried out in fetal development, particularly in the generation of critical metabolites such as methionine and SAM, which carry out roles in DNA methylation. The generation of these metabolites relies on methyltransferases in which Zn acts as a cofactor in transferring methyl groups to homocysteine (Uriu-Adams and Keen, 2010; Sanusi et al., 2021). The epigenetic modifications may, in turn, affect the expression and function of synaptic proteins.

The stimulation of proinflammatory cytokine production, including IL-1β through lipopolysaccharide exposure during development, has been shown to promote metallothionein to sequester Zn leading to further hypozincemia in the fetus (Kirsten et al., 2015). Thus, inflammation may affect synapses indirectly by restricting Zn availability. However, Zn itself plays a role in inflammation, particularly in deficient conditions such as malnutrition (Gammoh and Rink, 2017). A prominent pathology identified in ASD is the increase in pro-inflammatory signaling. Studies have found a significant increase in inflammatory markers such as IL-6, IL-8, and TNF-α in ASD (Alzghoul et al., 2019). Through a regulatory role in pro-inflammatory signaling pathways, Zn levels influence cytokine expression. In addition, Zn deficiency was shown to increase gut permeability in mice provoking inflammation in the brain, identified by an increase in IL-6 and GFAP (Sauer and Grabrucker, 2019). Decreased bioavailability of Zn and may lead to NLRP3-driven inflammation. This is also evident in Alzheimer’s diseases and leads to cognitive decline in animal models, which may translate to ASD models (Pellegrini et al., 2020; Rivers-Auty et al., 2021). Increased cytokine expression and the activation of glial cells have been linked to synapse loss and modification of synaptic function (Kim et al., 2020).

With approximately 20% of dietary Zn absorbed by intestinal bacteria, alterations in gut microbial composition occur in response to Zn availability (Reed et al., 2015; Koren and Tako, 2020). Excess dietary Zn has been positively implicated in resistance to *Clostridium difficile* (Zackular et al., 2016). In addition, altered microbial compositions were observed in Zn deficient conditions whereby the levels of Veruccomicrobia were decreased, and Firmicutes and Actinobacteria levels were increased. Actinobacteria are classed as pathobionts and have implications in various inflammatory diseases such as IBD. Thus, also through effects on microbiota, the metallome may alter the inflammatory state.

Lipid profiles in ASD are altered significantly through oxidative stress, gut microbiome disruption, nutrient intake, and so on (Tamiji and Crawford, 2010; Mazahery et al., 2017). Studies have indicated an imbalance in polyunsaturated fatty acids, which may contribute to ASD-like behaviors (Tamiji and Crawford, 2010). A recent study focused on the reduction of metal-containing proteins such as ceruloplasmin (Cu) and Transferrin (Fe) observed in individuals with ASD, which increased lipid peroxidation. However, further research is required to conclude whether this reduction is region-specific in the brain similar to Cu and Fe levels (Chauhan et al., 2004). Another study has used serum analysis to identify alterations in lipoproteins linking essential metal ratios in the process. Results concluded a significant correlation between Zn/Cu ratios with high-density lipoproteins and total cholesterol levels (Al-Bazzaz et al., 2020). Lipid metabolism and the metabolome play a critical role in cellular structures and remodeling in disease states. Disruption in the lipidome, such as increased cholesterol, also leads to the activation of the NLRP3 inflammasome giving rise to inflammatory pathologies (Anand, 2020). In turn, the gut microbiome, which has been shown to alter the inflammasome, can also contribute to the lipidome and *vice versa* (Fu et al., 2015; Wang et al., 2016).

The interlinked alterations across each “ome” can be traced back to metal dysregulation while also connecting and affecting further profiles resulting in the heterogeneity of ASD pathogenesis. However, to find an ASD-specific signature and possibly biomarkers for ASD, more research needs to investigate multiple “omes” in the same model or individual with ASD to allow correlation studies. Until then, studies investigating at least two “omes” may be used to generate a map of overlapping alterations.

### Correlating “Omics” Data for a Better Understanding of ASD

**FIGURE 2:**
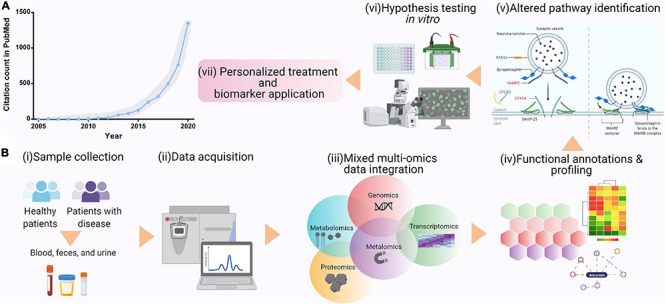
**(A)** Ever-growing popularity of multi-omics data analysis approach, shown as PubMed citation count from 2005 to 2020. **(B)** Mixed multi-omics data integration and analysis can pave the way for personalized medicine and biomarker discovery. This figure was created using Biorender.com.

ASD is a disorder with a multifactorial etiology and highly variable clinical phenotype, where thus far, 1,231 genes have been implicated in ASD (Simons foundation autism research initiative—SFARI). To fully uncover the causes of ASD data, scientists can utilize mixed multi-omics data integration approaches to discover a biological process that would be affected by alterations across the various “omes.” So far, this method has been successfully implemented in a variety of complex diseases, such as cancer (Liu et al., 2013, 2016; Muqaku et al., 2017). Mixed multi-omics is a systematic biological data analysis approach, where ≥ 2 data sets or “omes” derived from various analytical techniques, such as proteomics, genomics, transcriptomics, epigenomics, metabolomics, and microbiomics, are integrated and studied together (Figure 2). By fusing and integrating the multiple “omes,” scientists can view and analyze complex, extensive biological data, uncover novel associations between biological entities, and identify biomarkers that can be utilized for disease or developmental condition early diagnosis and treatment development.

Extracting meaningful information from each omics experiment with an extremely large number of data points is a computationally challenging task. This is further complicated by the vastly heterogeneous nature of biological data with non-linear interactions and shared effects on a multitude of factors, making it difficult to separate real biological signals from experimental noise. Noise may arise from the biological system being analyzed, the analytical platform used, and omics-specific analysis workflows. This challenge intensifies when mixed omics approaches are integrated together. The large-scale mixed multi-omics data integration requires careful data normalization, further statistical standardization, and even machine-learning tool application (Libbrecht and Noble, 2015; Min et al., 2017). Machine-learning tools are advantageous for analyzing amalgamated omics data and can carry out dimensionality reduction, clustering, the association of clinical measures, and disease prognosis (Li et al., 2018b).

Simple, graphical, and exploratory approaches such as principal component analysis (PCA) are often used to dimensionally reduce large data sets. In contrast, canonical correlation analysis (CCA) is utilized to study the overall correlation between two data sets. Additionally, other integrative frameworks for omics data utilize sparse CCA (Parkhomenko et al., 2009), multivariate partial least square analysis (Palermo et al., 2009), or multiple factor analysis (de Tayrac et al., 2009). Currently available tools for mixed multi-omics data integration can be partitioned into local and web-based applications, where local tools require R, Python, or Galaxy programming knowledge and are challenging to implement, these include mixOmics DIABLO/MINT, omicade4 or iOmicsPASS, and web-based applications such as, 3Omics, Paintomics, and Galaxy P/M which have user-friendly interfaces (Table 1). However, user-friendly web-based tools should only be used when the underlying methods are fully understood, as blind application often adversely affects progress in the field. At present, there is no single application or approach which can be applied for processing, analysis, and interpretation of data arising from integrated mixed multi-omics studies. Therefore, as computational biology is rapidly emerging, there is a need to develop amalgamated strategies and tools to aid in reproducible, high throughput, user-friendly, and effective platforms to study integrated mixed multi-omics data.

**TABLE 1:** Tool for mixed-omics data integration.

**Tools**	**Application**	**Working platform**
mixOmics—DIABLO (Singh et al., 2019b)	Mixed-omics data analysis and integration from same biological *N* samples measured on different “omics” platforms	R Bioconductor
mixOmics—MINT (Rohart et al., 2017)	Mixed-omics data analysis and integration of several independent data sets or studies measured on the same *P* predictors	R Bioconductor
omicade4 (Meng et al., 2014)	Multiple co-inertia analysis of multi-omics datasets	R Bioconductor
iOmicsPASS (Koh et al., 2019)	Network-based integration of multi-omics data for predictive subnetwork discovery	C + +
3Omics (Kuo et al., 2013)	Visualizes integrated human transcriptomic, proteomic, and metabolomic data	Web-based
Ingenuity pathway analysis, qiagen (Krämer et al., 2014)	Commercially available for integration and mapping of genomics, transcriptomics, proteomics, and metabolomics datasets	License, web, local
MoCluster (Meng et al., 2016)	Gene set analysis based on multiple omics data	R Bioconductor
MIMOmics (Auffray et al., 2016)	Integrated analysis of metabolomics, proteomics, glycomics, and genomic datasets in large studies	Web-based

## Conclusion

Taken together, combining results obtained from a multitude of individual “omics” studies may identify processes that lead to various disease states, but with one overlapping motif that may be the biological correlate of the shared features of ASD. With this knowledge, novel therapies could be developed targeting alternatives to the proteome, but with secondary effects on those protein networks currently in the focus of drug development in ASD (Park, 2020). An optimal starting point could include the metallome to counteract metal dyshomeostasis and, through its interaction with other “omes,” address symptoms emerging through effects of the microbiome, metabolome, lipidome, etc. Targeted metal supplementation or delivery of compounds targeting metal regulatory proteins may provide a critical instrument in developing therapeutic interventions due to the variety of roles of trace metals, for example, in histone modification, protein degradation, and inflammation (Chen et al., 2019). Given that metal dyshomeostasis has been identified in various diseases such as Alzheimer’s disease and Parkinson’s disease, those treatments may not be single-asset drugs. In addition, metal concentrations could provide valuable insight throughout pre-and early post-natal development as potential biomarkers of ASD since the metallome may serve as an indirect readout for alterations in the other, less accessible “omes.”

## Author Contributions

JS, SM, and AG drafted the manuscript. KM edited the manuscript. JS, SM, and AG prepared the figures. AG prepared the final version. All authors contributed to the article and approved the submitted version.

## Conflict of Interest

The authors declare that this study received funding from Zinpro Corp. The funder was not involved in the study design, collection, analysis, interpretation of data, the writing of this article, or the decision to submit it for publication.
