# Supplementary material for: The Metallome as a Link Between the “Omes” in Autism Spectrum Disorders
Source: Front Mol Neurosci. 2021 Jul 5;14:695873. doi: 10.3389/fnmol.2021.695873 (PMC8289253; doi:10.3389/fnmol.2021.695873)
Supplement: Supplementary file 1 [file Data_Sheet_1.PDF]

The Metallome as a link between the 'Omes' in Autism Spectrum Disorders

Janelle E. Stanton, Sigita Malijauskaite, Kieran McGourty, Andreas M. Grabrucker

Supplementary Table 1: On overview of "Omics" studies in ASD (Metallomics, Proteomics, Transcriptomics, Epigenomics, Metabolomics, Microbiomics, Inflammasomics)

Reference	Species	Method of analysis	Finding
**Metallomics**			
**1**	Human	Meta-analysis	↑Cu ↓Zn, Fe
**2**	Human	Meta-analysis	↑Pb, Hg
**3**	Human	Mass spectrometry	↑Cd ↓Zn, Cu, Mn, Cu
**4**	Human	ICP-Mass spectrometry	↑Al, Pb, Hg ↓Zn, Mg
**5**	Human	ICP-Mass spectrometry	↑Pb ↓Mn, Zn
**6**	Human	Inductively coupled plasma optical emission spectrometer	↑Pb, Hg, Cd ↓Zn, Mn
**7**	Human	Atomic absorption spectrometry	↑Cu, Pb, Hg ↓Zn, Mg
**8**	Human	ICP-Mass spectrometry	↑ ↓Ni, Cr,Al, Mn
**9**	Human	Atomic absorption spectrometry	↑Pb ↓Mn
**10**	Human	Meta-analysis	↓Ferritin , No significant difference in serum Fe
**11**	Human	Meta-analysis	↓Zn
**12**	Human	Meta-analysis	↓Zn/Cu
**13**	Human	ICP-Mass spectrometry	↑Cu ↓Zn
**14**	Human	Roentgen-fluorescence spectrometry	↑Cu, Pb, Hg, Cd ↓Zn, Mn
**15**	Human	Mass-spectrometry	↑Cu ↓Zn, Zn/Cu
**16**	Human	ICP-Atomic emission spectrometry	↑Zn, Fe, Ni, Cd, As
**17**	Human	Mass spectrometry	↑Zn, Fe, Mg ↓Cu
**18**	Human	Atomic absorption spectrometry	↑Cu, Pb, Hg ↓Zn, Al
**19**	Human	ICP-Mass spectrometry	↑Hg
**20**	Human	ICP-Mass spectrometry	↓Zn
**21**	Human	ICP-Mass spectrometry	↓Zn, Cu, Mg, Cr, Mn
**22**	Human	Atomic absorption spectrometry	↑Pb, Cu ↓Hg, Zn
**23**	Human	Atomic absorption spectrometry	↑Cu, Pb, Hg ↓Zn, Mg
**24**	Human	Spectrophotometry	↑Hg
**25**	Human	ICP-Mass spectrometry	↑Pb
**26**	Human	High-pressure liquid chromatography with fluorometric detection	↑Hg
**27**	Human	HPLC spectrofluorometry	↑Toxic metal markers (Porphyrin)
**28**	Human	Atomic absorption spectrometry	↑Hg, Pb, Cd ↓Cu, Mn, Fe
**29**	Human	Mass spectrometry	↑Hg, Pb
**30**	Human	Systemic analysis	↑Pb

**31**	Human	Case report, treatment for elevated Pb levels with chelating agent succimer	↓Pb levels during treatment
**Proteomics/Transcriptomics**			
**32**	Human	Blood biomarker analysis	↑IgD, suPAR, MAPK14, EPHB2, and DERM
**33**	Mouse	Illuminaseq.	↑ERK, RPLP1, RPL36A, DRD1
**34**	Mouse	Illumina seq.	↓mTORC1
**35**	Human	Metatranscriptomic analysis	↓parvalbumin, ↑TNF, HDAC1, GATA2
**36**	Human	LC-MS	↑Tubulin, MAPT, DLG4, APP, PSEN1, HDAC4, HTT
**37**	Mouse	Protein interaction/ Immunofluorescence	↑TLR4, Phospho-NFκB p65, IKKα, and IBA-1, iNOS ↓Arg-1
**38**	Human	Microarray analysis	↑IKKα ↓Tyk2, EIF4G1, PRKCI
**39**	Human	Protein expression	↑Alpha-2-macroglobulin, Alpha-1-antitrypsin, Haptoglobin, Fibrinogen, Transferrin, Prealbumin, Apolipoprotein A-I Apolipoprotein A-IV, Apolipoprotein J, Albumin
**40**	Human	Redox proteomics	↑C8 alpha chain, Ig kappa chain C
**41**	Human	LC-ESI-MS	↓Apo B-100 ↑Complement C1q subcomponent- C chain, Fibronectin 1, Complement factor H
**42**	Human	RP-HPLC-ESI-MS	↓Statherin, Histatin-1, Aprp, PRP-1, PRP-3
**43**	Human	MALDI-ToF-MS	↑Low molecular weight proteins such as serotonin, norepinephrin and neurotrophic factors
**44**	Human	Mass spectrometry	↑C3 complement protein
**45**	Human	Nano liquid chromatography-tandem mass spectrometry	↑PIP, LTF, annexin A1, neutrophil-defensin-1, lactoperoxidase, lipocalin-1 ↓salivary acidic proline-rich phospho-protein 1/2, submaxillary gland androgen-regulated protein 3B, antileukoproteinase, pleckstrin-homology domain-containing fam-ily H member, statherin
**46**	Human	NanoLC-MS/M	↑FRAT1, Kinesin family member 14, Integrin alpha6 subunit, growth hormone regulated TBC protein 1, parotid secretory protein, Prolactin-inducible protein precursor, Mucin-16, MRP14 ↓Alpha-amylase, CREB-binding protein, p532, Transferrin, Zn alpha2 glycoprotein, Zymogen granule protein 16, cystatin D, plasminogen
**47**	Human	MALDI–TOF–MS	↑Excretion of KNG-1, IgG1 heavy chain variable region, mannan-binding lectin serine protease-2 isoform-2 precursor
**48**	Human	NanoLC-MS/M	↑APOE, Serpina 1, FLBN1, FN1, C3, C5, AGT, VTN, Serpina 4, IGFALS ↓ACTN1, CALM1, CALR, ACTG1, PARVb, MAPRE2, ENO1, ITGA2B, FERMT3, EHD3, TLN1, VCL, VCP, THBS1
**49**	Human	MALDI-TOF MS	↑Serpina 5, platelet factor 4 (PF4), fatty acid binding protein 1(FABP1), apolipoprotein C-I precursor (APOC1), alpha-fetoprotein precursor (AFP), carboxypeptidase B2 (CPB2), trace amine-associated receptor 6 (TAAR6), and isoform1 of fibrinogen alpha chain precursor (FGA)
**50**	Human	SRM-MS	↑GFAP, CKB, SYN2, STBP1 ↓VIME, MAG, PLP1, STX1, SYT1, PACSIN1
**51**	Mouse	Microarray analysis	↑Stxbp1, Tom1l2, Agk, Gap43 ↓Rock2, Arl1,

**52**	Mouse	LC-MS/MS	↑MAP2, FKBP15, Snapin, TRF, MAP2 ↓MBP, UBE3A, MAP6, STXBP1
**53**	Rat	LC-MS/MS	MAPK, SUMO3, SNCA, PARK7 are candidate common hubs in Tuber-sclerosis complex
**54**	Mouse	SILAC	↑APC, FUS, tPA, SERBP, N-CAM↓Kcnma1α, VILIP, ARVCF
**55**	Mouse	LC-MS/MS	↑Auts2, Foxp1, core histone macro-H2A.2, Purα ↓Shank1-3, clusterin, alpha-1-antitrypsin 1, apolipoprotein A-I, HSPH1, HSP90α, HSP90β, HSPA4
**56**	Human	HPLC-MS/MS	↑Alterations present in Fibrinogen, Actin, Vinculin, Gelsolin
**57**	Mouse	LC–MS/MS	↑PSD95 ↓Synaptophysin, GluA1, GluA2, GluN1, GluN2B, GluK5
**58**	Mouse	LC-MS	Altered interactions of DLG, DLGAP and SHANK in postsynaptic density throughout development
**59**	Mouse	Agilent-014868 Whole Mouse Genome Microarray	En2-/- cerebellum: ↑immune response and major histocompatibility complex-related immunity. En2-/- hippocampus: ↓neurotransmission ↑seizures
**60**	Mouse	Affymetrix Mouse Genome 430 2.0 Array	Pten and Mecp2 knockdown lead to the most alterations in gene expression
**61**	Mouse	Affymetrix Mouse Gene 1.0 ST Array	CNV's in 16p11.2 result in dosage dependent alterations in 26 genes such as in *Gdpd3*
**62**	Mouse	Agilent-014868 Whole Mouse Genome Microarray	BTBR: ↑Microglial genes. En2−/− : ↑glutamatergic postsynaptic genes, FMRP-interacting genes and epilepsy-related genes
**63**	Mouse	Affymetrix Mouse Gene 1.1 ST Array	Altered expression of 116 in the nucleus accumbens and 251 genes in the medial prefrontal cortex. Many played roles in myelin functionality and stability.
**64**	Human	Published transcriptomic dataset analysis	For the ASD Convergent subtype, 13 distinct associated mRNA co-expression modules were identified along with 43 alternatively expressed miRNAs, 28 upregulated and 15 downregulated.
**65**	Mouse	Illumina HiSeq. Gene expression levels were quantified using featureCounts	Convergence on primarily neuronal (Nefh, Elavl4, Dclk, Cend1, Ina, and Chga) and mitochondrial (Atp5b, Slc25a3, Bcat1, Idh3a) genes was detected in three mouse models and compared to post-mortem human samples.
**66**	Human	Freeze 1 and 2 of the PsychENCODE Consortium dataset	Identification of neuronal and synaptic signaling genes and glial-immune or neuroinflammatory signals involving IFN-response, NFkB, astrocytes, and microglia as key alterations.
**67**	Human	Published transcriptomic dataset analysis	Genes related to mitochondrial function were differentially expressed in autism cerebral cortex and correlated with genes related to synaptic transmission.
**68**	Human	Illumina Ref8 v3 microarrays	444 genes with significant expression changes in ASD cortex and 2 genes in cerebellum implicating synaptic dysfunction, microglial, and immune dysregulation
**Epigenomics**			
**69**	Human	ChIP sequencing, RNA sequencing	↑Gene modules (MG4, MG5, MG51)
**70**	Human	Illumina & EWAS	455,068 CpG sites associated with ASD, 48 had suggestive significance
**71**	Human	ChIP sequencing, Fluorescence sorting	↑Histone H3 lysine 4 methylation and Histone H3 lysine 27 acetylation
**72**	Mouse	rt-PCR	Methylation of CpG sites↑Mid1,Nlgn1, Nf2, Nrgn1, Nrxn2, Neurod6, Efnb3, ↓Wnt3, Dlx1, Nlgn1, Nf2

**73**	Mouse	qrt-PCR	↑Methylation of promoter region in GAD1, GAD2 due to prenatal immune activation
**74**	Human	rt-PCR	↓Methylation of miR-142 promoter region
**75**	Human	Immunoprecipitation assays	↑Methylation of GAD1 promoter, no change observed in GAD2 and RELN promoters
**76**	Human	ChIP-sequencing	↑Acetylation of SLC30A5, CACNA1C, GRIN2B ↓HDA2, HDAC4, CX3CR1, FGFR2, CX3CR1, GRB10
**64**	Human	Meta-data analysis	A differential acetylation analysis found 2156 differentially acetylated regions at an FDR < 20%, identification of 3013 differentially methylated gene promoters, 2298 hypermethylated and 715 hypomethylated in ASD
**77**	Rat	RNA sequencing	17/37 MeCP-binding proteins are splicing factors, MeCP2 interacts with 5hmC and through epigenetic changes in histone markers can regulate mRNA splicing
**Metabolomics**			
**78**	Rat	NMR spectroscopy	Hippocampal conc. ↑NAA, Glu, and Gln, pyroglutamate, uracil, UFA, isoleucine, serine, tyrosine, phenylalanine, Lac, Asp, NA
			Cerebral conc. ↓phenylalanine, valine, alanine, glycine, isoleucine, glycerol
**79**	Human	Mass-spectrometry	Not gender specific
**80**	Human	NMR spectroscopy	↑Fumarate, Cis-aconitate, Carnitine, Glutamate (correlated with better social skills)
**81**	Human	Mass-spectrometry	↓7-Methylxanthine, Scylloinositol, Uric acid, Aminomalonic acid ↑Quinic acid, Hippuric acid, Tryptophan, 1-Methylhistidine, Cystine, Indole-3-acetic acid, Allylthioacetic acid, Leucine, Lactic acid
**82**	Human	Mass-spectrometry	Positive correlation identified between the level of Clostridium species and methyl esters (butanoic acid methyl ester, acetic acid methyl ester and pentanoic acid methyl ester) and indoles. Faecalibacterium, Ruminococcus and Bifidobact erium genera are positively correlated to the total SCFA. Bacteroides genus are correlated with total Free amino acids and propionic acid
**83**	Human	Flow-cytometric assay	↑COX-2, mPGES-1, PGE2
**Microbiomics**			
**84**	Human	Illumina seq.	↑ Acidobacteria,↓Firmicutes
**85**	Human	Pyrosequencing	↓Acidobacteria,↑Bacteroidetes, ↓Cyanobacteria, ↓Firmicutes, ↑Proteobacteria, ↑Tenericutes
**86**	Human	16S sequencing	↓Acidobacteria, ↑Bacteroidetes, ↑Proteobacteria
**87**	Mouse	Illumina seq.	↑ Acidobacteria, ↑Bacteroidetes
**88**	Mouse	Pyrosequencing	↑ Acidobacteria,↑Deferribacteres, ↑Firmicutes, ↑Tenericutes, ↓ Verrucomicrobia
**89**	Mouse	16S sequencing	↓Acidobacteria, ↑Bacteroidetes, ↓Firmicutes
**90**	Human	In silico analysis	↑Bacteroidetes
**91**	Human	Pyrosequencing	↓Bacteroidetes
**92**	Human	Pyrosequencing	↓Bacteroidetes
**93**	Human	Illumina seq.	↓Bacteroidetes, ↓Firmicutes
**94**	Mouse	Pyrosequencing	↓Bacteroidetes,↓Deferribacteres, ↑Firmicutes
**95**	Mouse	Illumina seq.	↑Bacteroidetes, ↓Cyanobacteria, ↓Firmicutes, ↑ Verrucomicrobia
**96**	Human	16S sequencing	↓Firmicutes

**97**	Mouse	16S sequencing	↑Proteobacteria
**Inflammasomics**			
**98**	Human	Meta-analysis	↓IL-10, IL-1 receptor antagonist. ↑IL-5. IFNα, IL-13
**99**	Human	Flow-cytometric assay	↓IFN-ɣ, IL-4, IL-10
**100**	Human	Luminex xMAP technology	↑IL-4, IL-10, TNFα, TNFβ
**101**	Human	qRT-PCR	↑IL-6
**82**	Human	Flow-cytometric assay	↑NFkB
**102**	Human	ELISA assay	↑IL-6, TNF
**103**	Human	Luminex cytokine assay	↑IL-6, IL-12
**104**	Human	RT-PCR array	↑AIM2 & NLRP3 inflammasomes, IL-1β, IL-18

Supplementary References

1. Saghazadeh, A., Ahangari, N., Hendi. K., Saleh. F., Rezaei, N. (2017). Status of essential elements in autism spectrum disorder: systematic review and meta-analysis. Rev Neurosci. 28(7):783–809. doi: 10.1515/revneuro-2017-0015

2. Saghazadeh, A., Rezaei, N. (2017) Systematic review and meta-analysis links autism and toxic metals and highlights the impact of country development status: Higher blood and erythrocyte levels for mercury and lead, and higher hair antimony, cadmium, lead, and mercury. Prog Neuropsychopharmacol Biol Psychiatry. 79(Pt B):340–68. doi: 10.1016/j.pnpbp.2017.07.011.

3. Fiore, M., Barone, R., Copat, C., Grasso, A., Cristaldi, A., Rizzo, R., et al. (2020) Metal and essential element levels in hair and association with autism severity. J Trace Elem Med Biol. 57:126409. doi: 10.1016/j.jtemb.2019.126409

4. Yasuda, H., Tsutsui, T., Suzuki, K. (2020) Metallomics Analysis for Assessment of Toxic Metal Burdens in Infants/Children and Their Mothers: Early Assessment and Intervention Are Essential. Biomolecules doi: 10.3390/biom11010006

5. Arora, M., Reichenberg, A., Willfors, C., Austin, C., Gennings, C., Berggren, S., et al. (2017) Fetal and postnatal metal dysregulation in autism. Nat Commun. 8(1):15493. doi: 10.1038/ncomms15493.

6. Qin, Y., Jian, B., Wu, C., Jiang, C., Kang, Y., Zhou, J., et al. (2018) A comparison of blood metal levels in autism spectrum disorder and unaffected children in Shenzhen of China and factors involved in bioaccumulation of metals. Environ Sci Pollut Res. 25(18):17950–6. doi: 10.1007/s11356-018-1957-7.

7. Lakshmi Priya, MD., Geetha, A. (2011) Level of Trace Elements (Copper, Zinc, Magnesium and Selenium) and Toxic Elements (Lead and Mercury) in the Hair and Nail of Children with Autism. Biol Trace Elem Res. 142(2):148–58. doi: 10.1007/s12011-010-8766-2

8. Skalny, A.V., Simashkova, N.V., Klyushnik, T.P., Grabeklis, A.R., Radysh, I.V., Skalnaya, M.G., et al. (2017) Assessment of serum trace elements and electrolytes in children with childhood and atypical autism. J Trace Elem Med Biol. 43:9–14. doi: 10.1016/j.jtemb.2016.09.009.

9. Hawari, I., Eskandar, M.B., Alzeer, S. (2020) The Role of Lead, Manganese, and Zinc in Autism Spectrum Disorders (ASDs) and Attention-Deficient Hyperactivity Disorder (ADHD): a Case-Control Study on Syrian Children Affected by the Syrian Crisis. Biol Trace Elem Res. 197(1):107–14. doi: 10.1007/s12011-020-02146-3

10. Tseng, P-T., Cheng, Y-S., Yen, C-F., Chen, Y-W., Stubbs, B., Whiteley, P., et al. (2018) Peripheral iron levels in children with attention-deficit hyperactivity disorder: a systematic review and meta-analysis. Sci Rep. 8(1):788. doi: 10.1038/s41598-017-19096-x.

11. Babaknejad, N., Sayehmiri, F., Sayehmiri, K., Mohamadkhani, A., Bahrami, S. (2016) The Relationship between Zinc Levels and Autism: A Systematic Review and Meta-analysis. Iran J Child Neurol. 10(4):1–9.

12. Sayehmiri, F., Babaknejad, N., Bahrami, S., Sayehmiri, K., Darabi, M., Rezaei-Tavirani, M. (2015) Zn/Cu Levels in the Field of Autism Disorders: A Systematic Review and Meta-analysis. Iran J Child Neurol. 9(4):1–9.

13. Russo, A.J., deVito, R. (2011) Analysis of Copper and Zinc Plasma Concentration and the Efficacy of Zinc Therapy in Individuals with Asperger’s Syndrome, Pervasive Developmental Disorder Not Otherwise Specified (PDD-NOS) and Autism. Biomark Insights. 6:BMI.S7286. doi: 10.4137/BMI.S7286

14. Tabatadze, T., Zhorzholiani, L., Kherkheulidze, M., Kandelaki, E., Ivanashvili, T. (2015) Hair heavy metal and essential trace element concentration in children with autism spectrum disorder. Georgian Med News. (248):77–82.

15. Li, S., Wang, J., Bjørklund, G., Zhao, W., Yin, C. (2014) Serum copper and zinc levels in individuals with autism spectrum disorders. Neuroreport. 25(15):1216–20. doi: 10.1097/WNR.0000000000000251

16. Vergani, L., Cristina, L., Paola, R., Luisa, A.M., Shyti, G., Edvige, V., et al. (2011) Metals, metallothioneins and oxidative stress in blood of autistic children. Res Autism Spectr Disord. 5(1):286–93.

17. Al-Farsi, Y.M., Waly, M.I., Al-Sharbati, M.M., Al-Shafaee, M.A., Al-Farsi, O.A., Al-Khaduri, M.M., et al. (2013) Levels of Heavy Metals and Essential Minerals in Hair Samples of Children with Autism in Oman: a Case–Control Study. Biol Trace Elem Res. 151(2):181–6. doi: 10.1007/s12011-012-9553-z.

18. Macedoni-Lukšič, M., Gosar, D., Bjørklund, G., Oražem, J., Kodrič, J., Lešnik-Musek, P., et al. (2015) Levels of Metals in the Blood and Specific Porphyrins in the Urine in Children with Autism Spectrum Disorders. Biol Trace Elem Res. 163(1):2–10. doi: 10.1007/s12011-014-0121-6

19. Geier, D.A., Kern, J.K., King, P.G., Sykes, L.K., Geier, M.R. (2012) Hair Toxic Metal Concentrations and Autism Spectrum Disorder Severity in Young Children. Int J Environ Res Public Health. 9(12):4486–97. doi: 10.3390/ijerph9124486.

20. Yasuda, H., Yoshida, K., Yasuda, Y., Tsutsui, T. (2011) Infantile zinc deficiency: Association with autism spectrum disorders. Sci Rep. 1(1):129. doi: 10.1038/srep00129.

21. Blaurock-busch, E., Amin, O.R., Rabah, T. (2011) Heavy Metals and Trace Elements in Hair and Urine of a Sample of Arab Children with Autistic Spectrum Disorder. Mædica. 6(4):247–57.

22. Elsheshtawy, E., Tobar, S., Sherra, K., Atallah, S., Elkasaby, R. (2011) Study of some biomarkers in hair of children with autism. Middle East Curr Psychiatry. 18(1):6–10. DOI:10.1097/01.XME.0000392842.64112.64

23. Lakshmi Priya, M.D., Geetha, A. (2011) Level of Trace Elements (Copper, Zinc, Magnesium and Selenium) and Toxic Elements (Lead and Mercury) in the Hair and Nail of Children with Autism. Biol Trace Elem Res. 142(2):148–58. doi:10.1007/s12011-010-8766-2.

24. Geier, D.A., Audhya, T., Kern, J.K., Geier, M.R. (2010) Blood mercury levels in autism spectrum disorder: Is there a threshold level? Acta Neurobiol Exp (Warsz). 70(2):177–86.

25. Clark, B., Vandermeer, B., Simonetti, A., Buka, I. (2010) Is lead a concern in Canadian autistic children? Paediatr Child Health. (1):17–22.

26. Geier, D.A., Geier, M.R. (2007) A Prospective Study of Mercury Toxicity Biomarkers in Autistic Spectrum Disorders. J Toxicol Environ Health A. 70(20):1723–30. doi: 10.1080/15287390701457712.

27. Nataf, R., Skorupka, C., Amet, L., Lam, A., Springbett, A., Lathe, R. (2006) Porphyrinuria in childhood autistic disorder: Implications for environmental toxicity. Toxicol Appl Pharmacol. 214(2):99–108. doi: 10.1016/j.taap.2006.04.008.

28. Al-Ayadhi, L.Y. (2005) Heavy metals and trace elements in hair samples of autistic children in central Saudi Arabia. Neurosci Riyadh Saudi Arab. 10(3):213–8.

29. Fido, A., Al-Saad, S. (2005) Toxic trace elements in the hair of children with autism. Autism Int J Res Pract. 9(3):290–8. doi: 10.1177/1362361305053255.

30. Filipek, P.A., Accardo, P.J., Baranek, G.T., Cook, E.H., Dawson, G., Gordon, B., et al. (1999) The Screening and Diagnosis of Autistic Spectrum Disorders. J Autism Dev Disord. 29(6):439–84. doi: 10.1023/a:1021943802493.

31. Eppright, T.D., Sanfacon, J.A., Horwitz, E.A. (1996) Attention deficit hyperactivity disorder, infantile autism, and elevated blood-lead: a possible relationship. Mo Med. 93(3):136–8.

32. Hewitson, L., Mathews, J.A., Devlin, M., Schutte, C., Lee, J., German, D.C. (2021) Blood biomarker discovery for autism spectrum disorder: A proteomic analysis. PLoS ONE 16(2). doi: 10.1371/journal.pone.0246581

33. Jin, C., Lee, Y., Kang, H., Jeong, K., Park, J., Zhang, Y., et al. (2021) Increased ribosomal protein levels and protein synthesis in the striatal synaptosome of Shank3-overexpressing transgenic mice. Mol Brain doi: 10.1186/s13041-021-00756-z.

34. Jin, C., Kang, H., Ryu, J.R., Kim, S., Zhang, Y., Lee, Y., et al. (2018) Integrative Brain Transcriptome Analysis Reveals Region-Specific and Broad Molecular Changes in Shank3-Overexpressing Mice. Front Mol Neurosci. 11:250. doi: 10.3389/fnmol.2018.00250.

35. Rahman, M.R., Petralia, M.C., Ciurleo, R., Bramanti, A., Fagone, P., Shahjaman, M., et al.(2020) Comprehensive Analysis of RNA-Seq Gene Expression Profiling of Brain Transcriptomes Reveals Novel Genes, Regulators, and Pathways in Autism Spectrum Disorder. Brain Sci 10(10). doi: 10.3390/brainsci10100747.

36. Abraham, J.R., Szoko, N., Barnard, J., Rubin, R.A., Schlatzer, D., Lundberg, K., et al. (2019) Proteomic Investigations of Autism Brain Identify Known and Novel Pathogenetic Processes. Sci Rep. 9(1):13118. doi: 10.1038/s41598-019-49533-y.

37. Xiao, L., Yan, J., Feng, D., Ye, S., Yang, T., Wei, H., et al. (2021) Critical Role of TLR4 on the Microglia Activation Induced by Maternal LPS Exposure Leading to ASD-Like Behavior of Offspring. Front Cell Dev Biol. 9:634837. doi: 10.3389/fcell.2021.634837

38. Shen, C., Zhao, X., Ju, W., Zou, X., Huo, L., Yan, W., et al. (2011) A Proteomic Investigation of B Lymphocytes in an Autistic Family: A Pilot Study of Exposure to Natural Rubber Latex (NRL) May Lead to Autism. J Mol Neurosci. 43(3):443–52. doi: 10.1007/s12031-010-9463-5.

39. Cortelazzo, A., Felice, C.D., Guerranti, R., Signorini, C., Leoncini, S., Zollo, G., et al. (2016) Expression and oxidative modifications of plasma proteins in autism spectrum disorders: Interplay between inflammatory response and lipid peroxidation. PROTEOMICS – Clin Appl. 10(11):1103–12. doi: 10.1002/prca.201500076.

40. Feng, C., Chen, Y., Pan, J., Yang, A., Niu, L., Min, J., et al. (2017) Redox proteomic identification of carbonylated proteins in autism plasma: insight into oxidative stress and its related biomarkers in autism. Clin Proteomics.14. doi: 10.1186/s12014-017-9138-0.

41. Corbett, B.A., Kantor, A.B., Schulman, H., Walker, W.L., Lit, L., Ashwood, P., et al. (2007) A proteomic study of serum from children with autism showing differential expression of apolipoproteins and complement proteins. Mol Psychiatry. 12(3):292–306. doi: 10.1038/sj.mp.4001943.

42. Castagnola, M., Messana, I., Inzitari, R., Fanali, C., Cabras, T., Morelli, A., et al. (2008) Hypo-Phosphorylation of Salivary Peptidome as a Clue to the Molecular Pathogenesis of Autism Spectrum Disorders. J Proteome Res. 7(12):5327–32. doi: 10.1021/pr8004088.

43. Taurines, R., Dudley, E., Conner, A.C., Grassl, J., Jans, T., Guderian, F., et al. (2010) Serum protein profiling and proteomics in autistic spectrum disorder using magnetic bead-assisted mass spectrometry. Eur Arch Psychiatry Clin Neurosci. 260(3):249–55. doi: 10.1007/s00406-009-0066-5.

44. Momeni, N., Bergquist, J., Brudin, L., Behnia, F., Sivberg, B., Joghataei, M.T., et al. (2012) A novel blood-based biomarker for detection of autism spectrum disorders. Transl Psychiatry. 2(3):e91–e91. doi: 10.1038/tp.2012.19.

45. Wetie, A.G.N., Wormwood, K.L., Russell, S., Ryan, J.P., Darie, C.C., Woods, A.G. (2015) A Pilot Proteomic Analysis of Salivary Biomarkers in Autism Spectrum Disorder. Autism Res. 8(3):338–50. doi: 10.1002/aur.1450

46. Wetie, A.G.N., Wormwood, K.L., Charette, L., Ryan, J.P., Woods, A.G., Darie, C.C. (2015) Comparative two-dimensional polyacrylamide gel electrophoresis of the salivary proteome of children with autism spectrum disorder. J Cell Mol Med. 19(11):2664–78. doi: 10.1111/jcmm.12658.

47. Suganya, V., Geetha, A., Sujatha, S. (2015) Urine proteome analysis to evaluate protein biomarkers in children with autism. Clin Chim Acta. 450:210–9. doi: 10.1016/j.cca.2015.08.015.

48. Shen, L., Zhang, K., Feng, C., Chen, Y., Li, S., Iqbal, J., et al. (2018) iTRAQ-Based Proteomic Analysis Reveals Protein Profile in Plasma from Children with Autism. PROTEOMICS – Clin Appl. 12(3):1700085. doi: 10.1002/prca.201700085.

49. Yang, J., Chen, Y., Xiong, X., Zhou, X., Han, L., Ni, L., et al. (2018) Peptidome Analysis Reveals Novel Serum Biomarkers for Children with Autism Spectrum Disorder in China. PROTEOMICS – Clin Appl. 12(5):1700164. doi: 10.1002/prca.201700164.

50. Broek, J.A., Guest, P.C., Rahmoune, H., Bahn, S. (2014) Proteomic analysis of post mortem brain tissue from autism patients: evidence for opposite changes in prefrontal cortex and cerebellum in synaptic connectivity-related proteins. Mol Autism. 5(1):41. doi: 10.1186/2040-2392-5-41.

51. Daimon, C.M., Jasien, J.M., Wood, W.H., Zhang, Y., Becker, K.G., Silverman, J.L., et al. (2015) Hippocampal Transcriptomic and Proteomic Alterations in the BTBR Mouse Model of Autism Spectrum Disorder. Front Physiol. 6. doi: 10.3389/fphys.2015.00324

52. Wei, H., Ma, Y., Liu, J., Ding, C., Hu, F., Yu, L. (2016) Proteomic analysis of cortical brain tissue from the BTBR mouse model of autism: Evidence for changes in STOP and myelin-related proteins. Neuroscience. 312:26–34. doi: 10.1016/j.neuroscience.2015.11.003.

53. Niere, F., Namjoshi, S., Song, E., Dilly, G.A., Schoenhard, G., Zemelman, B.V., et al. (2016) Analysis of Proteins That Rapidly Change Upon Mechanistic/Mammalian Target of Rapamycin Complex 1 (mTORC1) Repression Identifies Parkinson Protein 7 (PARK7) as a Novel Protein Aberrantly Expressed in Tuberous Sclerosis Complex (TSC)*. Mol Cell Proteomics. 15(2):412–30. doi: 10.1074/mcp.M115.055079.

54. Liao, L., Park, S.K., Xu, T., Vanderklish, P., Yates, J.R. (2008) Quantitative proteomic analysis of primary neurons reveals diverse changes in synaptic protein content in fmr1 knockout mice. Proc Natl Acad Sci U S A. 105(40):15281–6. doi: 10.1073/pnas.0804678105.

55. Pacheco, N.L., Heaven, M.R., Holt, L.M., Crossman, D.K., Boggio, K.J., Shaffer, S.A., et al. (2017) RNA sequencing and proteomics approaches reveal novel deficits in the cortex of Mecp2-

deficient mice, a model for Rett syndrome. Mol Autism. 8(1):56. doi: 10.1186/s13229-017-0174-4

56. Kaysheva, A.L., Stepanov, A.A., Kopylov, A.T., Butkova, T.V., Pleshakova, T., Ryabtsev, V.V., et al. (2019) Pilot data of serum proteins from children with autism spectrum disorders. Data Brief. 27:104558.

57. Reim, D., Distler, U., Halbedl, S., Verpelli, C., Sala, C., Bockmann, J., et al. (2017) Proteomic Analysis of Post-synaptic Density Fractions from Shank3 Mutant Mice Reveals Brain Region Specific Changes Relevant to Autism Spectrum Disorder. Front Mol Neurosci.10. doi: 10.3389/fnmol.2017.00026.

58. Li, J., Zhang, W., Yang, H., Howrigan, D.P., Wilkinson, B., Souaiaia, T., et al. (2017) Spatiotemporal profile of postsynaptic interactomes integrates components of complex brain disorders. Nat Neurosci. 20(8):1150–61. doi: 10.1038/nn.4594.

59. Sgadò, P., Provenzano, G., Dassi, E., Adami, V., Zunino, G., Genovesi, S., et al. (2013) Transcriptome profiling in engrailed-2 mutant mice reveals common molecular pathways associated with autism spectrum disorders. Mol Autism. 4(1):51. doi: 10.1186/2040-2392-4-51.

60. Lanz, T.A., Guilmette, E., Gosink, M.M., Fischer, J.E., Fitzgerald, L.W., Stephenson, D.T., et al. (2013) Transcriptomic analysis of genetically defined autism candidate genes reveals common mechanisms of action. Mol Autism. 4(1):45. doi: 10.1186/2040-2392-4-45.

61. Horev, G., Ellegood, J., Lerch, J.P., Son, Y-E.E., Muthuswamy, L., Vogel, H., et al. (2011) Dosage-dependent phenotypes in models of 16p11.2 lesions found in autism. Proc Natl Acad Sci. 108(41):17076–81. doi: 10.1073/pnas.1114042108.

62. Provenzano, G., Corradi, Z., Monsorno, K., Fedrizzi, T., Ricceri, L., Scattoni, M.L., et al. (2016) Comparative Gene Expression Analysis of Two Mouse Models of Autism: Transcriptome Profiling of the BTBR and En2−/− Hippocampus. Front Neurosci. 10. doi: 10.3389/fnins.2016.00396.

63. Richetto, J., Chesters, R., Cattaneo, A., Labouesse, M.A., Gutierrez, A.M.C., Wood, T.C., et al. (2017) Genome-Wide Transcriptional Profiling and Structural Magnetic Resonance Imaging in the Maternal Immune Activation Model of Neurodevelopmental Disorders. Cereb Cortex. 27(6):3397–413. doi: 10.1093/cercor/bhw320.

64. Ramaswami, G., Won, H., Gandal, M. J., Haney, J., Wang, J. C., Wong, C. C. Y., et al. (2020). Integrative genomics identifies a convergent molecular subtype that links epigenomic with transcriptomic differences in autism. Nat. Commun. 11,4873. doi:10.1038/s41467-020-18526-1.

65. Gordon, A., Forsingdal, A., Klewe, I.V., Nielsen, J., Didriksen, M., Werge, T., et al. (2021). Transcriptomic networks implicate neuronal energetic abnormalities in three mouse models harboring autism and schizophrenia-associated mutations. *Mol. Psychiatry* 26(5):1520-1534. doi: 10.1038/s41380-019-0576-0.

66. Gandal, M.J., Zhang, P., Hadjimichael, E., Walker, R.L., Chen, C., Liu, S., et al. (2018). Transcriptome-wide isoform-level dysregulation in ASD, schizophrenia, and bipolar disorder. *Science* 362(6420). doi: 10.1126/science.aat8127.

67. Schwede, M., Nagpal, S., Gandal, M.J., Parikshak, N.N., Mirnics, K., Geschwind, D.H., et al. (2018). Strong correlation of downregulated genes related to synaptic transmission and mitochondria in post-mortem autism cerebral cortex. *J. Neurodev. Disord.* 10(1):18. doi: 10.1186/s11689-018-9237-x.

68. Voineagu, I., Wang, X., Johnston, P., Lowe, J.K., Tian, Y., Horvath, S., et al. (2011). Transcriptomic analysis of autistic brain reveals convergent molecular pathology. *Nature* 474(7351):380-4. doi: 10.1038/nature10110.

69. Amiri, A., Coppola, G., Scuderi, S., Wu, F., Roychowdhury, T., Liu, F., et al. (2018) Transcriptome and epigenome landscape of human cortical development modeled in brain organoids. Science. 362(6420). doi: 10.1126/science.aat6720.

70. Andrews, S.V., Sheppard, B., Windham, G.C., Schieve, L.A., Schendel, D.E., Croen, L.A., et al. (2018) Case-control meta-analysis of blood DNA methylation and autism spectrum disorder. Mol Autism. 9:40. doi: 10.1186/s13229-018-0224-6.

71. Shulha, H.P., Cheung, I., Whittle, C., Wang, J., Virgil, D., Lin, C.L., et al. (2012) Epigenetic signatures of autism: trimethylated H3K4 landscapes in prefrontal neurons. Arch Gen Psychiatry. 69(3):314–24. doi: 10.1001/archgenpsychiatry.2011.151.

72. Richetto, J., Massart, R., Weber-Stadlbauer, U., Szyf, M., Riva, M.A., Meyer, U. (2017) Genome-wide DNA Methylation Changes in a Mouse Model of Infection-Mediated Neurodevelopmental Disorders. Biol Psychiatry. 81(3):265–76. doi: 10.1016/j.biopsych.2016.08.010.

73. Labouesse, M.A., Dong, E., Grayson, D.R., Guidotti, A., Meyer, U. (2015) Maternal immune activation induces GAD1 and GAD2 promoter remodeling in the offspring prefrontal cortex. Epigenetics. 10(12):1143–55. doi: 10.1080/15592294.2015.1114202.

74. Mor, M., Nardone, S., Sams, D.S., Elliott, E. (2015) Hypomethylation of miR-142 promoter and upregulation of microRNAs that target the oxytocin receptor gene in the autism prefrontal cortex. Mol Autism. 6:46. doi: 10.1186/s13229-015-0040-1.

75. Zhubi, A., Chen, Y., Guidotti, A., Grayson, D. (2017) Epigenetic regulation of RELN and GAD1 in the frontal cortex (FC) of autism spectrum disorder (ASD) subjects. Int J Dev Neurosci Off J Int Soc Dev Neurosci. 62:63–72. doi: 10.1016/j.ijdevneu.2017.02.003.

76. Sun, W., Poschmann, J., Cruz-Herrera Del Rosario, R., Parikshak, N.N., Hajan, H.S., Kumar, V., et al. (2016) Histone Acetylome-wide Association Study of Autism Spectrum Disorder. Cell. 167(5):1385-1397.e11. doi: 10.1016/j.cell.2016.10.031.

77. Cheng, T-L., Chen, J., Wan, H., Tang, B., Tian, W., Liao, L., et al. (2017) Regulation of mRNA splicing by MeCP2 via epigenetic modifications in the brain. Sci Rep. 7(1):42790. doi: 10.1038/srep42790.

78. Abreu, A.C., Navas, M.M., Fernández, C.P., Sánchez-Santed, F., Fernández, I. (2021) NMR-Based Metabolomics Approach to Explore Brain Metabolic Changes Induced by Prenatal Exposure to Autism-Inducing Chemicals. ACS Chem Biol. doi: 10.1021/acschembio.1c00053.

79. Courraud, J., Ernst, M., Svane Laursen, S., Hougaard, D.M., Cohen, A.S. (2021) Studying Autism Using Untargeted Metabolomics in Newborn Screening Samples. J Mol Neurosci. doi: 10.1007/s12031-020-01787-2.

80. Sotelo-Orozco, J., Abbeduto, L., Hertz-Picciotto, I., Slupsky, C.M. (2020) Association Between Plasma Metabolites and Psychometric Scores Among Children With Developmental Disabilities: Investigating Sex-Differences. Front Psychiatry. 11:579538. doi: 10.3389/fpsyt.2020.579538.

81. Mussap, M., Siracusano, M., Noto, A., Fattuoni, C., Riccioni, A., Rajula, H.S.R., et al. (2020) The Urine Metabolome of Young Autistic Children Correlates with Their Clinical Profile Severity. Metabolites 10(11). doi: 10.3390/metabo10110476.

82. De Angelis, M., Piccolo, M., Vannini, L., Siragusa, S., De Giacomo, A., Serrazzanetti, D.I., et al. (2013) Fecal Microbiota and Metabolome of Children with Autism and Pervasive Developmental Disorder Not Otherwise Specified. PLoS ONE. 8(10). doi: 10.1371/journal.pone.0076993.

83. Qasem, H., Al-Ayadhi, L., Bjørklund, G., Chirumbolo, S., El-Ansary, A. (2018) Impaired lipid metabolism markers to assess the risk of neuroinflammation in autism spectrum disorder. Metab Brain Dis. 33(4):1141–53. doi: 10.1007/s11011-018-0206-6.

84. Liu, S., Li, E., Sun, Z., Fu, D., Duan, G., Jiang, M., et al. (2019) Altered gut microbiota and short chain fatty acids in Chinese children with autism spectrum disorder. Sci Rep. 9(1):287. doi: 10.1038/s41598-018-36430-z.

85. Finegold, S.M., Dowd, S.E., Gontcharova, V., Liu, C., Henley, K.E., Wolcott, R.D., et al. (2010) Pyrosequencing study of fecal microflora of autistic and control children. Anaerobe. 16(4):444–53. doi: 10.1016/j.anaerobe.2010.06.008.

86. Coretti, L., Paparo, L., Riccio, M.P., Amato, F., Cuomo, M., Natale, A., et al. (2018) Gut Microbiota Features in Young Children With Autism Spectrum Disorders. Front Microbiol. 9. doi: 10.3389/fmicb.2018.03146.

87. Liu, F., Horton-Sparks, K., Hull, V., Li, R.W., Martínez-Cerdeño, V. (2018) The valproic acid rat model of autism presents with gut bacterial dysbiosis similar to that in human autism. Mol Autism. 9(1):61. doi: 10.1186/s13229-018-0251-3.

88. Sauer, A.K., Grabrucker, A.M. (2019) Zinc Deficiency During Pregnancy Leads to Altered Microbiome and Elevated Inflammatory Markers in Mice. Front Neurosci. 13. doi: 10.3389/fnins.2019.01295.

89. Sgritta, M., Dooling, S.W., Buffington, S.A., Momin, E.N., Francis, M.B., Britton, R.A., et al. (2019) Mechanisms Underlying Microbial-Mediated Changes in Social Behavior in Mouse Models of Autism Spectrum Disorder. Neuron. 101(2):246-259.e6. doi: 10.1016/j.neuron.2018.11.018.

90. Maigoro, A.Y., Lee, S. (2021) Gut Microbiome-Based Analysis of Lipid A Biosynthesis in Individuals with Autism Spectrum Disorder: An In Silico Evaluation. Nutrients. 13(2). doi: 10.3390/nu13020688.

91. Strati, F., Cavalieri, D., Albanese, D., De Felice, C., Donati, C., Hayek, J., et al. (2017) New evidences on the altered gut microbiota in autism spectrum disorders. Microbiome. 5(1):24. doi: 10.1186/s40168-017-0242-1.

92. Williams, B.L., Hornig, M., Buie, T., Bauman, M.L., Paik, M.C., Wick, I., et al. (2011) Impaired Carbohydrate Digestion and Transport and Mucosal Dysbiosis in the Intestines of Children with Autism and Gastrointestinal Disturbances. PLOS ONE. 6(9):e24585. doi: 10.1371/journal.pone.0024585.

93. Cao, X., Liu, K., Liu, J., Liu, Y-W., Xu, L., Wang, H., et al. (2021) Dysbiotic Gut Microbiota and Dysregulation of Cytokine Profile in Children and Teens With Autism Spectrum Disorder. Front Neurosci. 15. doi: 10.3389/fnins.2021.635925.

94. de Theije, C.G.M., Wopereis, H., Ramadan, M., van Eijndthoven, T., Lambert, J., Knol, J., et al. (2014) Altered gut microbiota and activity in a murine model of autism spectrum disorders. Brain Behav Immun. 37:197–206. doi: 10.1016/j.bbi.2013.12.005.

95. Golubeva, A.V., Joyce, S.A., Moloney, G., Burokas, A., Sherwin, E., Arboleya, S., et al. (2017) Microbiota-related Changes in Bile Acid & Tryptophan Metabolism are Associated with Gastrointestinal Dysfunction in a Mouse Model of Autism. EBioMedicine. 24:166–78. doi: 10.1016/j.ebiom.2017.09.020.

96. Zurita, M.F., Cárdenas, P.A., Sandoval, M.E., Peña, M.C., Fornasini, M., Flores, N., et al. (2020) Analysis of gut microbiome, nutrition and immune status in autism spectrum disorder: a case-control study in Ecuador. Gut Microbes. 11(3):453–64. doi: 10.1080/19490976.2019.1662260.

97. Coretti, L., Cristiano, C., Florio, E., Scala, G., Lama, A., Keller, S., et al. (2017) Sex-related alterations of gut microbiota composition in the BTBR mouse model of autism spectrum disorder. Sci Rep. 7(1):45356.

98. Saghazadeh, A., Ataeinia, B., Keynejad, K., Abdolalizadeh, A., Hirbod-Mobarakeh, A., Rezaei, N. (2019) Anti-inflammatory cytokines in autism spectrum disorders: A systematic review and meta-analysis. Cytokine. 123:154740. doi: 10.1016/j.cyto.2019.154740.

99. Abdallah, M.W., Larsen, N., Mortensen, E.L., Atladóttir, H.Ó., Nørgaard-Pedersen, B., Bonefeld-Jørgensen, E.C., et al. (2012) Neonatal levels of cytokines and risk of autism spectrum disorders: An exploratory register-based historic birth cohort study utilizing the Danish Newborn Screening Biobank. J Neuroimmunol. 252(1):75–82. doi: 10.1016/j.jneuroim.2012.07.013.

100. Abdallah, M.W., Larsen, N., Grove, J., Nørgaard-Pedersen, B., Thorsen, P., Mortensen, E.L., et al. (2013) Amniotic fluid inflammatory cytokines: Potential markers of immunologic dysfunction in autism spectrum disorders. World J Biol Psychiatry doi: 10.3109/15622975.2011.639803.

101. Abruzzo, P.M., Matté, A., Bolotta, A., Federti, E., Ghezzo, A., Guarnieri, T., et al. (2019) Plasma peroxiredoxin changes and inflammatory cytokines support the involvement of neuro-inflammation and oxidative stress in Autism Spectrum Disorder. J Transl Med. 17. doi: 10.1186/s12967-019-2076-z.

102. Tsilioni, I., Taliou, A., Francis, K., Theoharides, T.C. (2015) Children with autism spectrum disorders, who improved with a luteolin-containing dietary formulation, show reduced serum levels of TNF and IL-6. Transl Psychiatry. 5(9):e647–e647. doi: 10.1038/tp.2015.142.

103. Ashwood, P., Krakowiak, P., Hertz-Picciotto, I., Hansen, R., Pessah, I., Water, J.V (2011) Elevated plasma cytokines in autism spectrum disorders provide evidence of immune dysfunction and are associated with impaired behavioral outcome. Brain Behav Immun. 25(1):40. doi: 10.1016/j.bbi.2010.08.003

104. Saresella, M., Piancone, F., Marventano, I., Zoppis, M., Hernis, A., Zanette, M., et al. (2016) Multiple inflammasome complexes are activated in autistic spectrum disorders. Brain Behav Immun. 57:125–33. doi: 10.1016/j.bbi.2016.03.009.
